# A Study on Childhood Trauma and Sexual Narcissism in Individuals with Compulsive Sexual Behavior Receiving Counseling

**DOI:** 10.1007/s10508-025-03137-y

**Published:** 2025-05-05

**Authors:** Rotem Yaakov, Aviv Weinstein

**Affiliations:** 1 Department of Criminology, Ariel University, Ariel, Israel; 2 Department of Psychology, The Isador and Ruth Kastin Chair for Brain Research, Ariel University, Science Park, 40700 Ariel, Israel

**Keywords:** Compulsive sexual behavior disorder, Childhood physical and mental abuse, Sexual abuse, Sexual narcissism

## Abstract

Compulsive sexual behavior (CSB) has been associated with trauma and neglect in childhood. There is evidence that CSB is related to child physical and sexual abuse. Sexual narcissism was linked with aggression, sex at a young age, and many partners for sex. This study examined the associations between childhood abuse and neglect, sexual narcissism, and hypersexual behavior among participants of a sex addiction support group and a group of control participants. Participants were 118 adults, including 72 men and 46 women, with a mean age of 32 years (SD = 9.32) and an age range of 18–59. The sample included 59 participants in the sex addiction group (sexaholics anonymous, SA), 36 men and 23 women with a mean age 31.41 years (SD = 8.13), and the control group from the general population included 36 men and 23 women with a mean age 32.47 years (SD = 10.42). Questionnaires included a sociodemographic questionnaire, the Hypersexual Behavior Inventory, the Sexual Narcissism Scale, and the Childhood Trauma Questionnaire. Childhood trauma was associated with sexual narcissism and with hypersexual behavior. A mediation model showed that sexual narcissism and child trauma significantly contributed to ratings of hypersexual behavior, and explained 60.3% of the variance of hypersexual behavior ratings. In addition, using the Bootstrapping method, the indirect effects found in this model showed that sexual narcissism increased the likelihood of hypersexual behavior, and it was a significant mediating factor between trauma and hypersexual behavior. In summary, this study indicates that sexual narcissism mediated the relationships between childhood trauma and hypersexual behavior. These findings explain the role of sexual narcissism and CSA in hypersexual behavior and it may have clinical implications for the treatment of CSB disorder.

## Introduction

Compulsive sexual behavior disorder (CSBD) represents a maladaptive and excessive sexual behavior, persisting despite adverse consequences and despite efforts to stop them (Levine, 2010). CSB, or hypersexual behavior, involves uncontrolled sexual behavior and has clinical, economic and social consequences (Karila et al., 2014; Kraus et al., 2016, 2018; Weinstein et al., 2015a, 2015b). Previous studies have investigated causes of CSB and its development (Dhuffar & Griffiths, 2014; Lewczuk et al., 2017). There are several studies showing associations between personality and CSB (Egan & Parmar, 2013; Grubbs et al., 2015; Schmitt, 2004; Shimoni et al., 2018). These studies measured personality dimensions that are typically investigated through models such as the Big Five model of personality.

The World Health Organization (2020) defines child maltreatment as “all forms of physical and emotional ill treatment, sexual abuse, neglect, and exploitation that result in actual or potential harm to the child’s health, development or dignity.” Child abuse is related to physical and mental health problems, and it is a risk factor for CSB. Surveys have reported a high incidence of sexual abuse in childhood among those with CSB (Black et al., 1997; Carnes & Delmonico, 1996). CSA severity can predict later CSB (Blain et al., 2012; Kuzma & Black, 2008).

Narcissistic personality disorder involves an exaggerated perception of self-importance, exploitation and a lack of empathy, and a constant need to be admired (American Psychiatric Association, 2013). Sexuality is an important component of narcissistic personality disorder. Narcissists may respond aggressively to rejections they may experience in sexual relations, and they are attracted to sexual relationships due to their need for positive regard and admiration. They have exaggerated views of their sexual abilities and false beliefs that their victims want these sexual activities despite their protests (Baumeister et al., 2002). Narcissists have low levels of empathy and high levels of hostility and exploitation, and they may force sexual relations they desire despite their harmful aggression (Raskin et al., 1991). Finally, individuals with narcissistic personality disorder may concentrate on their own pleasure and may not pay attention to their partner's needs, and they can be very controlling and manipulative in their sexual relationships expecting submission from their partners.

Narcissism has been associated with CSB in participants of sex anonymous support groups (Efrati et al., 2019) and among young adults in Germany (Jepsen & Brzank, 2022). The Narcissistic Personality Inventory (NPI) (Raskin & Terry, 1988) is a global tool that does not directly address individuals in sexual situations. Mischel and Shoda (1995) have argued therefore that sexual aggression is triggered in narcissistic men during sexual situations where they lack empathy. The NPI is insensitive to these triggers and does not differentiate between sexual and non-sexual narcissists and therefore is not successful in predicting sexual aggression (Baumeister et al., 2002). Widman and McNulty (2010) have argued that it is important to use measures of narcissistic personality which is specific to sexual behaviors. They have therefore proposed the term sexual narcissism, which is a psychological or psycho-sexual term that relates to sexual aggression and its cognitive components (e.g., a lack of empathy, a sense of entitlement). They have provided and validated an assessment tool for sexual narcissism that would be more appropriate than a global assessment tool. Sexual narcissism was associated with aggression and with intercourse with many partners and sex at a young age (Widman & McNulty, 2010). Sexual narcissism may be viewed as a spectrum or continuum that is not constrained to individuals with narcissistic personality disorder.

We have recently shown that CSB is related to child sexual abuse, physical abuse, and neglect and mental abuse and sex addiction among women who utilized applications for finding sexual partners (Ashkenazi et al., 2022). Ratings of abuse (physical neglect, sexual abuse, and physical and mental abuse) have explained a high percentage (57%) of the variance of ratings of sexual addiction. There is a further need to investigate whether personality characteristics mediate the association between CSA and CSB. Although general personality factors only account for a small percentage of variance of CSB (Shimoni et al., 2018), the more specific psychological or psychosexual construct of sexual narcissism may have a more important role in mediating this association.

The aim of this study was therefore to investigate the relationships between childhood trauma and neglect, sexual narcissism, and hypersexual behavior in individuals seeking psychological support for CSB and healthy control participants. Childhood trauma and sexual narcissism were hypothesized to be positively linked with hypersexual behavior. We also predicted that childhood trauma and sexual narcissism would contribute to the variance of ratings of hypersexual behavior. Since Widman and McNulty (2010) have argued that sexual narcissism is an important trigger of sexual aggression, we hypothesized that it could be a potential mediator between early childhood trauma and hypersexual behavior. We have therefore predicted that sexual narcissism would mediate the relationships between childhood trauma and hypersexual behavior.

## Method

### Participants

There were 118 participants in the study. Inclusion criteria were healthy men and women at age range of 18–59. Exclusion criteria were standard exclusion criteria that are used in clinical studies such as psychiatric diagnosis or history of neurological conditions and infectious disease (HIV AIDS). Half of the participants were classified by the researchers as CSB, and half were classified as non-CSB following criteria defined by Reid et al. (2011) as a score above 53 on the Hypersexual Behavior Inventory (HBI). The mean age of the participants with CSB was 31.41 (SD = 8.13), the mean age of the participants who were not CSB was 32.47 (SD = 10.42), and the age difference between them was not significant *z* = −0.07, *p* = 0.942. The CSB group consisted of 59 participants, 36 men and 23 women, who participated in a support group for sex addiction (SA). The control group included 36 men and 23 women.

### Measures

#### Demographic Questionnaire

The demographic self-report questionnaire included questions on age, education, gender, religiosity, sexual orientation, and marital status.

### Procedure

For the CSB group, questionnaires were distributed to participants in a “sexaholics anonymous” (SA) support group and other support groups for sex addiction. Control participants were recruited by advertising online in social networking forums. Participants answered questionnaires online. They were informed that the study investigates sexual behavior for research purpose, and they are maintained anonymous. The control sample was recruited by the researchers by using social networks, and it was matched by the researchers with the CSB clinical group.

#### Hypersexuality and Compulsive Sexual Behavior

Although hypersexuality and CBSD are different labels referring to the same phenomena, those different terms reflect different theoretical frameworks for the understanding of excessive sexual behavior (Sassover & Weinstein, 2020). Some view problematic or excessive sexual behavior as a feature of hypersexual disorder. Although it was proposed by Kafka (2010) as a new psychiatric disorder called Hypersexual Disorder for consideration in the DSM-5, the American Psychiatric Association (2013) did not include it in the DSM-5. Concerns were raised about the lack of biological, epidemiological, and neuropsychological testing and the absence of a clear distinction between normal and pathological levels of sexual desires and behaviors (see Kraus et al., 2016 for review).

#### Hypersexual Behavior Inventory

The Hypersexual Behavior Inventory (HBI) questionnaire has specific items that describe using sex as a coping strategy. Results from studies using the HBI show significant differences between patient groups and control groups on this scale (Reid & Carpenter, 2009; Reid et al., 2009), and a substantive body of research shows that CSBD patients use sex to deal with unwanted experiences or situations (Gilliland et al., 2011).

The HBI questionnaire (Reid et al., 2011) includes 19 items. Ratings were on a five-point scale from 1 "never" to 5 "very often." Scores range from 19 to 95. Scores are divided into three factors: (1) control, (2) coping, and (3) consequences. Previous studies have shown high internal consistency of the HBI (Cronbach's *α* = 0.95), and for the three scales: control of sex (Cronbach's *α* = 0.94), hypersexual behavior (Cronbach's *α* = 0.90), and handling emotional experiences (Cronbach's *α* = 0.87) (Reid et al., 2011). In our study, the internal consistency of the three factors was: control of sex (Cronbach's *α* = 0.98), hypersexual behavior (Cronbach's *α* = 0.97), and handling emotional experiences (Cronbach's *α* = 0.95) and overall Cronbach's *α* = 0.98.

#### Sexual Narcissism Questionnaire

The Sexual Narcissism Scale (SNS) was validated on two groups of college students (299 participants, including 152 men and 147 women in the first sample and 363 men in the second sample) who enrolled in psychology courses at a large southeastern university in the US by Widman and McNulty (2010).

The SNS has 20 items. Items were self-rated on a five-point scale from 1 "strongly disagree" to 5 "strongly agree." Scores range from 20 to 100. The questionnaire has four scales: Sexual Exploitation, Sexual Entitlement, Low Sexual Empathy, and Sexual Skill. Initial reliability tests showed high internal consistency measures among the overall scores (Cronbach’s *α* = 0.91) and within each scale: Sexual Exploitation *α* = 0.87, Sexual Entitlement *α* = 0.81, Low Sexual Empathy *α* = 0.77, and Sexual Skill *α* = 0.76 (Widman & McNulty, 2010). In our study, internal consistency for Sexual Exploitation was *α* = 0.79, Sexual Entitlement *α* = 0.86, Low Sexual Empathy *α* = 0.85, and Sexual Skill *α* = 0.82) and overall Cronbach's *α* = 0.90.

#### Childhood Trauma Questionnaire

The Childhood Trauma Questionnaire (Bernstein et al., 1994) contains 28 items and 6 scales, including physical abuse (*n* = 5), emotional neglect (*n* = 7), sexual abuse (*n* = 5), emotional abuse (*n* = 5), physical neglect (*n* = 3), and denial of the experience of abuse (*n* = 4). One item (number 4) was for both physical neglect and denial for the experience of abuse. The items were rated on a scale from 1 "never happened" to 5 "very often happened." Scores range from 28 to 140, and there are 10 reversed items. Cronbach's *α* of the Hebrew version domains (Tomarkin, 2007) ranged from 0.82 to 0.94; in the current study internal consistency for physical abuse was 0.86, mental abuse = 0.90, sexual abuse = 0.90, physical neglect = 0.58, emotional neglect = 0.93, and overall Cronbach's *α* = 0.98.

### Data Analysis

Results were analyzed on version 21 of the Statistical Package for Social Science (SPSS) (IBM Corp. Armonk, NY, USA). Parametric/nonparametric group comparisons were used based on normality tests. Pearson's chi-squared test was used to compare demographic factors such as marital status, education, age and hypersexual behavior ratings. Mann–Whitney tests compared ratings of sexual narcissism and childhood trauma measures between the CSB groups and the control group. The Spearman correlation test was used for simple correlations between the study's variables. A parallel moderation model analysis of childhood trauma, sexual narcissism, and hypersexual behavior was performed using the 'lavaan' package in the PROCESS macro for SPSS (Hayes, 2015).

### Power Calculations

Power calculation of the mediation model was conducted by using G*Power 3.1.9.4 (Faul et al., 2009) based on a multiple variable regression model with a mediating effect size.

## Results

Normality tests (Shapiro and Anderson–Darling tests) of sexual narcissism, childhood trauma, hypersexual behavior, and age found that the variables were not normally distributed. Thus, the statistics tests used in the paper were nonparametric tests and those that did not assume the normality of the variables. To test whether there was a significant group difference in demographic variables (gender, religiosity, family status, academic education, and sexuality), chi-square tests for independence were used. Table 1 shows a comparison between groups in all demographic variables in all participants M (SD) (*n* = 118).

**Table 1:** Demographic questionnaire ratings and chi-square test for differences between study groups in gender, religiosity, family status, academic education, and sexuality (*n* = 118)

			Group		
Variables	Categories		CSB	Non-CSB	*χ*^2^
Gender	Women	*n*	23	23	
		%	39.0%	39.0%	
	Men	*n*	36	36	*χ*^2^(1) = 0.00, *p* = 1.00
		%	61.0%	61.0%	*φ* = 0
Religious	No	*n*	33	51	
		%	55.9%	86.4%	
	Yes	*n*	26	8	*χ*^2^(1) = 13.39, *p* < .001
		%	44.1%	13.6%	*φ* = 0.34
Family status	In a relationship	*n*	28	32	
		%	49.1%	54.2%	
	Single	*n*	23	20	
		%	40.4%	33.9%	
	Divorced/separated	*n*	6	7	*χ*^2^(2) = 0.52, *p* = .77
		%	10.5%	11.9%	*V* = 0.05
Academic education	No	*n*	31	21	
		%	52.5%	35.6%	
	Yes	*n*	28	38	*χ*^2^(1) = 3.44, *p* = .06
		%	47.5%	64.4%	*φ* = 0.17
Sexuality	Bisexual	*n*	13	2	
		%	22.0%	3.4%	
	Homosexual	*n*	10	12	
		%	16.9%	20.3%	
	Heterosexual	*n*	36	45	*χ*^2^(2) = 9.25, *p* = .01
		%	61.1%	76.3%	*V* = 0.20

*CSB* compulsive sexual behavior

As seen in Table 1, significant group differences were shown between the CSB group and non-CSB groups in religiosity and sexuality. Thus, 44% of the participants in the CSB group were religious, while only 14% of the participants in the non-CSB group were religious. About 22% of the participants in the CSB group were bisexual, and only 61% were heterosexual, while only 4% of the participants in the non-CSB group were bisexual, and 76% were heterosexual.

### Compulsive Sexual Behavior and Non-Compulsive Sexual Behavior Comparison in Measures of Sexual Narcissism, Childhood Trauma, and Hypersexual Behavior

To test whether there were significant differences between participants who were classified as CSB and those who were classified as non-CSB on all questionnaires, Mann–Whitney tests were conducted. As can be seen from Table 2, participants with CSB scored higher on measures of sexual narcissism, childhood trauma, and hypersexual behavior than those who were not classified as CSB. See Table 2 for mean scores (SD) and range on questionnaires and group differences in all participants M (SD) (*n* = 118); effect sizes are provided in the table.

**Table 2:** Differences between CSB and non-CSB participants in sexual narcissism, childhood trauma, and hypersexual behavior (*n* = 118)

Variables	CSB	*n*	Mean item score	SD	*Z*
SNS	Yes	59	3.07	0.72	
	No	59	2.09	0.48	*Z* = −6.93, *p* < 0.001, *r* = 0.64
CTQ	Yes	59	2.41	0.79	
	No	59	1.51	0.53	*Z* = −6.34, *p* < 0.001, *r* = 0.58
HBI	Yes	59	4.02	0.87	
	No	59	1.50	0.46	*Z* = −9.37, *p* < 0.001, *r* = 0.86

*CSB* compulsive sexual behavior, CTQ Childhood Trauma Questionnaire, HBI Hypersexual Behavior Inventory, SNS Sexual Narcissism Questionnaire

### Sex Differences

To test whether there were sex differences in sexual narcissism, childhood trauma and hypersexual behavior, Mann–Whitney tests were conducted. As can be seen from Table 3, males scored higher on hypersexual behavior measures than females, but not on sexual narcissism and childhood trauma measures. Effect sizes are provided in the table.

**Table 3:** Sex differences in sexual narcissism, childhood trauma, and hypersexual behavior (*n* = 118)

Variables	Gender	*n*	*M*	*SD*	*Z*
SNS	Female	46	2.45	0.82	
	Male	72	2.67	0.75	*Z* = −1.68, *p* = 0.09, *r* = 0.16
CTQ	Female	46	1.94	0.90	
	Male	72	1.98	0.74	*Z* = −0.97, *p* = 0.33, *r* = 0.09
HBI	Female	46	2.38	1.24	
	Male	72	3.00	1.51	*Z* = −2.39, *p* = 0.02, *r* = 0.23

*CTQ* Childhood Trauma Questionnaire, *HBI* Hypersexual Behavior Inventory, *SNS* Sexual Narcissism Questionnaire

### Religiosity

To test whether there were significant differences between religious and non-religious participants in sexual narcissism, childhood trauma, and hypersexual behavior, Mann–Whitney tests were conducted. As can be seen from Table 4, among CSB participants, religious participants scored higher on sexual narcissism and hypersexual behavior but not on childhood trauma compared with non-religious participants. Effect sizes are provided in the table.

**Table 4:** Comparison between religious and non-religious participants in sexual narcissism, childhood trauma, and hypersexual behavior (*n* = 118)

Variables	Religious	*n*	*M*	*SD*	*Z*
SNS	No	84	2.44	0.71	
	Yes	34	2.93	0.84	*Z* = −2.88, *p* = 0.01*, r* = 0.28
CTQ	No	84	1.91	0.84	
	Yes	34	2.10	0.70	*Z* = −1.78, *p* = 0.08*, r* = 0.17
HBI	No	84	2.31	1.17	
	Yes	34	3.89	1.44	*Z* = −4.92, *p* < 0.001, *r* = 0.47

*CTQ* Childhood Trauma Questionnaire

*HBI* Hypersexual Behavior Inventory

*SNS* Sexual Narcissism Questionnaire

### Differences Between Heterosexual and Non-Heterosexual Participants

To test whether there were significant differences between heterosexual, homosexual or bisexual participants in sexual narcissism, childhood trauma and hypersexual behavior, Kruskal–Wallis tests were conducted. As can be seen from Table 5, bisexual participants scored higher on hypersexual measures than homosexual and heterosexual participants. Effect sizes are provided in the table.

**Table 5:** Differences between heterosexual, homosexual or bisexual participants in sexual narcissism, childhood trauma, and hypersexual behavior (*n* = 118)

Variables	Status	*n*	*M*	*SD*	*H*
SNS	Bisexual	15	3.01	0.88	
	Homosexual	22	2.43	0.62	
	Heterosexual	81	2.54	0.78	*H*(2) = 4.25, *p* = 0.119, *η*^2^ = 0.021
CTQ	Bisexual	15	2.18	0.80	
	Homosexual	22	2.05	0.80	
	Heterosexual	81	1.90	0.80	*H*(2) = 3.17, *p* = 0.205, *η*^2^ = 0.011
HBI	Bisexual	15	3.74	1.15	
	Homosexual	22	2.68	1.26	
	Heterosexual	81	2.60	1.48	*H*(2) = 8.28, *p* = 0.016, *η*^2^ = 0.060

*CTQ* Childhood Trauma Questionnaire, *HBI* Hypersexual Behavior Inventory, *SNS* Sexual Narcissism Questionnaire

### Relationship Status

To test whether there were significant differences between participants who are in a relationship, single or separated in sexual narcissism, childhood trauma, and hypersexual behavior, Kruskal–Wallis tests were conducted. There were no significant group differences in sexual narcissism *H*(2) = 1.61, *p* = 0.45, *η*^2^ = 0.00, childhood trauma *H*(2) = 2.96, *p* = 0.23, *η*^2^ = 0.01 or hypersexual behavior *H*(2) = 0.19, *p* = 0.91, *η*^2^ = 0.00 measures.

### Academic and Non-Academic Status

To test whether there were significant differences between academic and non-academic participants in sexual narcissism, childhood trauma, and hypersexual behavior, Mann–Whitney tests were conducted. Non-academic participants scored higher on sexual narcissism, *Z* = −3.00, *p* = 0.003, *r* = 0.29, childhood trauma, *Z* = −2.78, *p* = 0.01, *r* = 0.27, and hypersexual behavior, *Z* = −2.78, *p* = 0.01, *r* = 0.27, than academic participants.

### The Associations Between Childhood Trauma, Sexual Narcissism, and Hypersexual Behavior

To test whether there are relationships between sexual narcissism, childhood trauma and hypersexual behavior, for the entire sample and the two groups separately, spearman correlations were used (see Table 6). For the entire sample, all the correlations were positive and significant. Thus, the higher the sexual narcissism, the higher the childhood trauma and the hypersexual behavior. For the participants who were classified as CSB, the correlations between hypersexual behavior and sexual narcissism and between hypersexual behavior and childhood trauma were positive and significant. Thus, the higher the hypersexual behavior, the higher the sexual narcissism and childhood trauma.

**Table 6:** Spearman correlations between the study questionnaires for the entire sample and the two groups separately (*n* = 118)

Variables	Sexual narcissism	Childhood trauma	Hypersexual behavior
Combined			
SNS			
CTQ	0.54**		
HBI	0.73**	0.62**	
CSB Group			
SNS			
CTQ	0.25		
HBI	0.43**	0.31*	
Non-CSB Group			
SNS	0.31*		
CTQ	0.59**	0.26	
HBI			

*CSB* compulsive sexual behavior, *CTQ* Childhood Trauma Questionnaire, HBI Hypersexual Behavior Inventory, *SNS* Sexual Narcissism Questionnaire

^*^p < 0.05; **p < 0.0;1 ***p < 0.001

For the participants who were classified as non-CSB, the correlations between sexual narcissism and childhood trauma and between sexual narcissism and hypersexual behavior were positive and significant. Thus, the higher the sexual narcissism, the higher the childhood trauma and hypersexual behavior.

### Predicting Hypersexual Behavior

To test whether the study variables predict hypersexual behavior, hierarchic regression was conducted. In the first step, gender, religiosity, education and sexuality were entered into the regression. In the second step, the group variable was added, and in the third step, childhood trauma and sexual narcissism were entered into the regression. To handle the regression with variables that were not normally distributed, we used robust standard errors to test for significance, and we tested the normality of the residuals that were found to be normally distributed.

As can be seen in Table 7, the first step was statistically significant. Religiosity, education and the difference between bisexuality and heterosexuality were significant. The second step was also significant, and the group factor was found to be significant. However, gender was also significant, but education and the difference between bisexuality and heterosexuality were not significant. In the last step, childhood trauma and sexual narcissism were found to be significant.

**Table 7:** Hierarchic regression for predicting hypersexual behavior (*n* = 118)

Step	Variables	*B*	*SE.B*	*ß*	*R*^2^	$\Delta {R}^{2}$
1	Male	0.13	0.28	0.04		
	Religious	1.37***	0.34	0.43		
	Education	− 0.51*	0.24	− 0.18		
	Bisexual–Heterosexual	0.82**	0.27	0.9		
	Homosexual–Heterosexual	0.09	0.36	0.02	28.6%^***^	28.6%^***^
2	Male	0.47***	0.14	0.16		
	Religious	0.49**	0.17	0.15		
	Education	− 0.22	0.12	− 0.08		
	Bisexual–Heterosexual	0.05	0.17	0.01		
	Homosexual–Heterosexual	− 0.09	0.17	− 0.02		
	Group	2.32***	0.14	0.81	83.3%^***^	54.7%^***^
3	Male	0.36**	0.12	0.12		
	Religious	0.54***	0.15	0.17		
	Education	− 0.07	0.12	− 0.02		
	Bisexual–Heterosexual	0.05	0.16	0.01		
	Homosexual–Heterosexual	− 0.06	0.15	− 0.02		
	Group	1.76***	0.16	0.61		
	CTQ	0.25**	0.08	0.14		
	SNS	0.34**	0.11	0.19	87.2%^***^	3.9%^***^

*CTQ* Childhood Trauma Questionnaire; *SNS* Sexual Narcissism Questionnaire

^*^p < 0.05 **p < 0.01 ***p < 0.001

Thus, males scored higher in hypersexual behavior than females. Religious people scored higher on hypersexual behavior than non-religious participants. Non-academic participants scored higher on hypersexual behavior than academic participants. Bisexual participants scored higher on hypersexual behavior than homosexual participants. Participants in the CSB group scored higher on hypersexual behavior than the non-CSB control group. And lastly, the higher the childhood trauma and sexual narcissism, the higher the hypersexual behavior.

### The Association Between Childhood Trauma and Hypersexual Behavior

To test whether sexual narcissism mediates the association between childhood trauma and hypersexual behavior, we used the 'lavaan' package in R software, using WLS as an estimator that does not assume the normality of the variables. Due to the size of the sample, it is not possible to test a separate model for each study group. However, the correlations between the study variables for the general sample and for each sample separately show that there is a similar trend of relationships between the study variables in the two samples. Moreover, there is no significant difference between the correlations for the two samples (*p* > 0.05). Therefore, it can be assumed that the model is not substantially different between the two research groups.

**Fig. 1:**
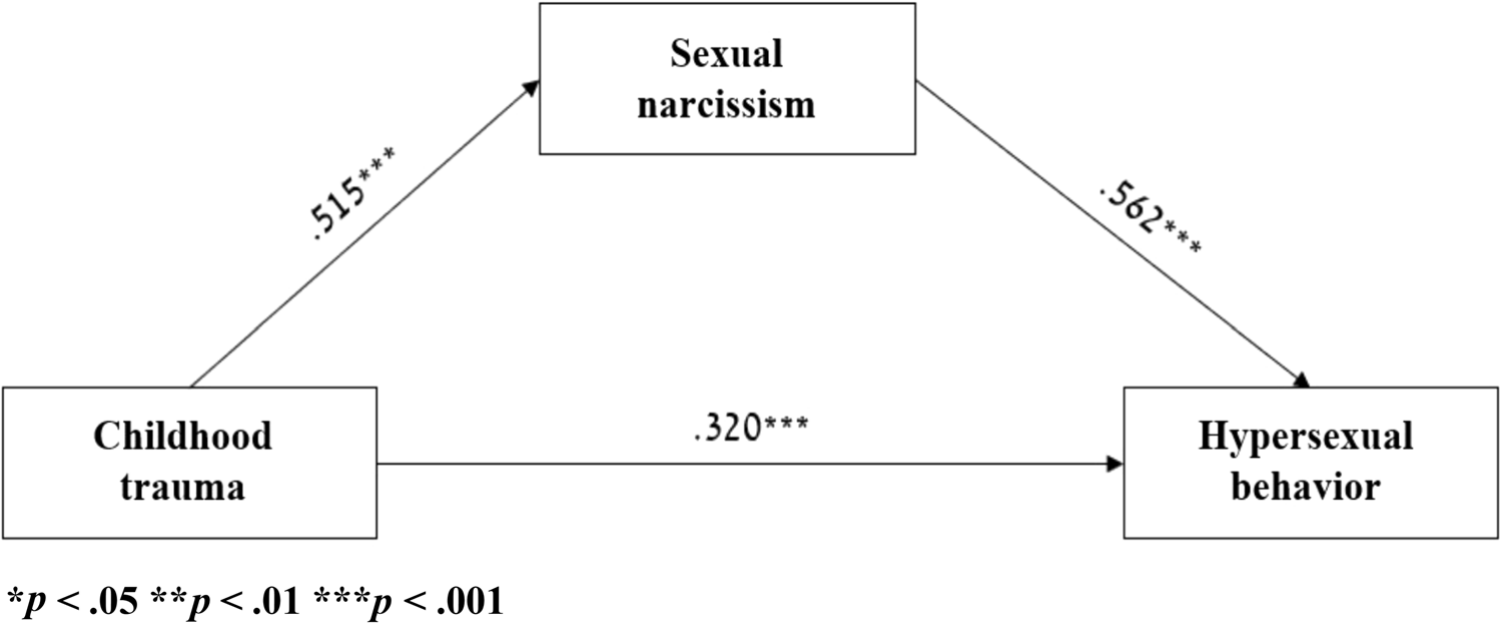
Mediation model between childhood trauma and hypersexual behavior (*n* = 118)

Results indicate that sexual narcissism was predicted positively by childhood trauma, and hypersexual behavior was predicted positively by sexual narcissism and childhood trauma (see Table 8 and Fig. 1). To test the significance of the indirect effects in the analysis using the bootstrapping technique, 5,000 resamples were used to generate 95% confidence intervals. Indirect effects in which zero is not included in the 95% *CI* indicate a significant effect at *α* < 0.05. Tests of the indirect effects of childhood trauma on hypersexual behavior via sexual narcissism were significant [*B* = 0.518, *SE* = 0.12, CI 0.29–0.75]. Thus, higher childhood trauma predicts higher sexual narcissism, which, in turn, predicted higher hypersexual behavior. The model was significant and it explained 60.3% of hypersexual behavior.

**Table 8:** Mediation between childhood trauma and hypersexual behavior via sexual narcissism (*n* = 118)

Predicted variable	Predicting variable	B	SE	LLCI	ULCI
SNS	CTQ	0.50	0.08	0.34	0.66
HBI	CTQ	0.58	0.15	0.29	0.86
	SNS	1.04	0.15	0.75	1.33

*CTQ* Childhood Trauma Questionnaire, *HBI* Hypersexual Behavior Inventory, *SNS* Sexual Narcissism Questionnaire

## Discussion

This results of this study indicated that childhood trauma and sexual narcissism were associated with hypersexual behavior and they contributed to ratings of hypersexual behavior in participants in a support group for CSB and control participants. Furthermore, sexual narcissism mediated the association between childhood trauma and hypersexual behavior. The study has also confirmed previous evidence for higher sex addiction scores in men (Eisenman et al., 2004; Levi et al., 2020; Simoni et al., 2018; Weinstein et al., 2015a, 2015b).

Previous studies showed that high rates of childhood trauma and abuse in those seeking treatment for sex addiction, particularly among women who showed higher rates and severity of trauma than men (Opitz et al., 2009; Perera et al., 2009; Ross, 1996; Schwartz & Southern, 2000; Tedesco & Bola, 1997; Weiss, 2000). Childhood sexual abuse severity is a predictor of CSB in adulthood as well as for depression and anxiety (Blain et al., 2012). Perera et al. (2009) showed that sexual abuse experiences and poor family relationships during childhood were related to vulnerability to the initiation and maintenance of uncontrolled sexual behaviors. Males with a child sexual abuse history are more likely to develop sexual addiction and may need long-term treatment.

Child sexual abuse was associated with negative attitudes toward sex and sexual dysfunctional behavior (Vaillancourt-Morel et al., 2015). CSA is also a risk for post-traumatic stress disorder (PTSD), depression and anxiety in particular in female adolescents (Adams et al., 2018). CSB as well as bingeeating can be an avoidance mechanism from these disorders (Blain et al., 2012; Coleman et al., 2003; Turner, 2008). Sexual avoidance and compulsivity are evident in victims of sexual abuse, manifested by attachment problems, dissatisfaction from relationships and mood swings. Vaillancourt-Morel et al. (2015) have reported that CSA was positively associated with CSB and sexual avoidance. Sexual compulsivity and sexual avoidance predicted impaired perception of their relationship with an intimate partner (Vaillancourt-Morel et al., 2015). Finally, CSB was associated with attachment problems in childhood (Hal, 2013). Individuals with avoidant or anxious attachment showed fear of intimacy and substituted fantasy and sexual behavior with intimacy (Zapf et al., 2008). Anxious and avoidant attachment were associated with CSB (Weinstein et al., 2015a, 2015b). Difficulties in attachment in early childhood can make individuals use addiction as comfort whereas childhood trauma may make it almost impossible for children to form healthy attachment bonds and it may lead to CSB in adulthood (Hall, 2013). These studies demonstrate that PTSD, depression, anxiety, and attachment may affect the association between CSA and CSB.

Previous studies have shown a relationship between CSB and narcissism in individuals diagnosed with narcissistic personality disorder (Coleman et al., 2003), students and peers (Kasper et al., 2015) and among those who attend sex anonymous support groups (Efrati et al., 2019). Sexual behavior can manifest narcissistic traits like desire for power and admiration, exploitation and sense of entitlement, and having many partners may facilitate these traits. As mentioned earlier, narcissistic men show sexual aggression in sexual situations which activate their sexual desires and lack of empathy for sexual partners (Mischel & Shoda, 1995). Due to the limitations of global measures of narcissism there was a need to assess the situations that give rise to sexual narcissism, such as sexual aggression among men. There is evidence that sexual narcissists make great efforts to find sexual partners and they face a higher risk of rejection and consequent aggression. They may often have distorted perceptions of sexuality and relationships, a tendency to ignore rejection from others or may be indifferent to others feelings (Baumeister et al., 2000, 2002). Covert narcissism in dating men correlated with physical assault and sexual narcissism and correlated with partner sexual coercion (Ryan et al., 2008). Narcissistic sense of entitlement and exploitation of others are related to sexual aggression whereas narcissistic grandiosity is negatively related to sexual aggression (Zeigler-Hill et al., 2013). The sexual narcissism scale (Widman & McNulty, 2010) that has been used in our study addresses the issue of sexual narcissism by examining the ingredients of narcissism such as grandiose beliefs, a sense of entitlement, a lack of empathy and manipulations or exploitations that are being used in sexual situations.

There are few studies on the association between CSB and other personality traits. Black et al. (1997) reported frequent paranoid, histrionic, obsessive–compulsive and passive-aggressive personality disorders in their sample. Shimoni et al. (2018) showed that personality variables have only explained a small percentage (11.7%) of the variance of sex addiction scores. Openness to experience and neuroticism positively correlated with sex addiction scores, whereas conscientiousness negatively correlated with sex addiction scores. Other factors than personality can explain CSB, including internal and external factors (Griffiths (2017), mental distress (Grubbs et al., 2015) and sexual arousal (Rettenberger et al., 2016). Our results indicate that sexual narcissism incorporates both narcissistic personality and behavioral factors such as aggression and fear of rejection in individuals with CSB. The relationship between CSB and sexual narcissism, especially regarding male aggressive behavior may have important implications for the work of forensic and psychological research and treatment. Treatment programs for compulsive sexual behavior should include sexual narcissism and aggression into psychological treatment.

Finally, this study has indicated that males scored higher in hypersexual behavior than females. The evidence for sex differences in CSB was supported by previous studies (Corley & Hook, 2012; Laier & Brand, 2014; Weinstein et al., 2015a, 2015b). The results also showed that religious people scored higher on hypersexual behavior than non-religious participants. This is supported by previous evidence that religious adolescents are higher in CSB than secular ones in Israel (Efrati, 2019). Jennings et al. (2021) have reviewed the literature and found small to moderate positive relationships between religiosity and CSB. Finally, bisexual participants scored higher on hypersexual behavior than homosexual participants. A smallscale study has found that lesbian women had higher rates of sexual compulsivity than heterosexual women whereas there was no difference in sexual compulsivity between homosexual and heterosexual men (Weinstein et al., 2015a, 2015b). Similar to the findings that non-academic participants scored higher on hypersexual behavior than academic participants, these findings should be viewed with caution and require replication in larger samples.

### Limitations

This study was cross-sectional, and it was based on self-reports and it could be biased due to social desirability. No causal relationships can be inferred and longitudinal studies are highly recommended.

The sample of this study was predominantly Jewish (95.8%) and, therefore, it has some cross-cultural limitations in terms of its generalization. Other potential limitations arise from scale selection, additional confounding variables, alternative statistical analyses or other methodological considerations.

### Conclusions

This study showed that childhood trauma (abuse and neglect) and the personality trait of sexual narcissism have contributed to ratings of hypersexual behavior in individuals seeking help for CSB. These findings could explain the role of sexual narcissism, and childhood trauma in the development of hypersexual behavior. The study could contribute to treatment programs for compulsive sexual behavior by including personality traits such as sexual narcissism into psychological treatment.

## Data Availability

The data that support the findings of this study are available from the authors upon request. Restrictions apply to the availability of these data, which were used under license for the current study and so are not publicly available. The data are, however, available from the authors upon reasonable request.
